# Intracellular Calcium Deficits in *Drosophila* Cholinergic Neurons Expressing Wild Type or FAD-Mutant *Presenilin*

**DOI:** 10.1371/journal.pone.0006904

**Published:** 2009-09-04

**Authors:** Kinga Michno, David Knight, Jorge M. Campussano, Diana van de Hoef, Gabrielle L. Boulianne

**Affiliations:** 1 Program in Developmental and Stem Cell Biology, The Hospital for Sick Children, Toronto, Ontario, Canada; 2 Department of Molecular Genetics, University of Toronto, Toronto, Ontario, Canada; 3 Department of Cell and Molecular Biology, Faculty of Biological Sciences, Catholic University of Chile, Santiago, Chile; 4 Department of Anatomy and Neurobiology, University of California Irvine, Irvine, California, United States of America; University of Cambridge, United Kingdom

## Abstract

Much of our current understanding about neurodegenerative diseases can be attributed to the study of inherited forms of these disorders. For example, mutations in the *presenilin 1* and *2* genes have been linked to early onset familial forms of Alzheimer's disease (FAD). Using the *Drosophila* central nervous system as a model we have investigated the role of *presenilin* in one of the earliest cellular defects associated with Alzheimer's disease, intracellular calcium deregulation. We show that expression of either wild type or FAD-mutant *presenilin* in *Drosophila* CNS neurons has no impact on resting calcium levels but does give rise to deficits in intracellular calcium stores. Furthermore, we show that a loss-of-function mutation in *calmodulin*, a key regulator of intracellular calcium, can suppress *presenilin*-induced deficits in calcium stores. Our data support a model whereby *presenilin* plays a role in regulating intracellular calcium stores and demonstrate that *Drosophila* can be used to study the link between *presenilin* and calcium deregulation.

## Introduction

Alzheimer's disease (AD) is a neurodegenerative disorder characterized clinically by progressive dementia and histopathologically by the formation of neuritic plaques, neurofibrillary tangles (NFT) and ultimately neuronal cell death. Despite being the most prevalent and intensely studied form of dementia there is still no effective cure for AD. Although the majority of AD cases are sporadic, 5–10% are familial (FAD) and inherited in an autosomal dominant fashion. Approximately 50% of FAD cases have been attributed to mutations in three genes, amyloid precursor protein (APP) [1], presenilin-1 (PSEN1) [2] or presenilin-2 (PSEN2) [3].

Presenilins are integral membrane proteins synthesized within the endoplasmic reticulum (ER) as full-length holoproteins. In the ER, presenilins undergo proteolytic cleavage generating N- and C-terminal fragments, which remain associated. Along the secretory pathway, presenilins associate with presenilin enhancer-2, nicastrin and anterior pharynx defective-1. Together these proteins constitute the γ-secretase complex. This complex has proteolytic activity and is known to cleave several type I transmembrane proteins including Notch and APP. APP proteolysis is particularly important to AD because aberrant APP proteolysis results in the deposition of Aβ fragments, which are the primary components of neuritic plaques. While Aβ deposition is a cellular hallmark of AD, it remains unclear whether or not this process is the primary cause of AD. Neurodegeneration in the absence of plaque formation [4]–[7] suggests that other *presenilin*-induced toxic cellular processes may compromise neuronal function independent of Aβ generation and ultimately set the stage for the onset of AD pathogenesis. In fact, AD etiology is believed to involve several aberrant cellular processes including protein aggregation, oxidative stress as well as intracellular calcium deregulation.

Deregulation of intracellular calcium signaling is an early event in AD pathogenesis and precedes any symptoms [8]. More specifically, internal calcium stores including the endoplasmic reticulum (ER) and Golgi apparatus have been reported to be either under or, over-loaded in cells expressing FAD-mutant forms of PS1 [9]–[12] or PS2 [10], [13]. The apparent discrepancies in published results may be attributed to the use of different FAD-presenilin mutations, different cell types (often non-neuronal) and different experimental approaches. It is clear that further studies focused on understanding the role of presenilins in intracellular calcium dysfunction are needed to resolve these inconsistencies.

Changes in cytosolic calcium concentration normally function as a second messenger system mediating a wide range of cellular processes, many of which are relevant to AD etiology including learning and memory as well as cell death. Internal calcium stores play an important role in facilitating intracellular calcium homeostasis by regulating calcium release and storage. The ER contains two main types of calcium release channels, the ryanodine receptor (RyR) and the inositol 1,4,5-triphosphate receptors (IP_3_R). Presenilins have been shown to physically interact with both of these channels and to influence their activity [14]–[17]. Presenilins have also been shown to physically interact with a number of known transducers of calcium signaling including calmyrin [18], [19], sorcin [20] and calsenilin [11], [21]. Finally, one study has suggested that presenilins themselves may function as passive ER calcium channels [22]. Despite all the evidence linking presenilin function to intracellular calcium homeostasis, the precise mechanisms by which presenilins regulate calcium dynamics remain unresolved.

In this study we have investigated the impact of wild type and mutant presenilin expression on intracellular calcium dynamics in primary *Drosophila* cholinergic neurons. Importantly, unlike most presenilin studies performed in *Drosophila,* our work focuses on presenilin function specifically in the fly central nervous system (CNS). The genetic tractability of *Drosophila melanogaster* makes this organism an ideal model to study the function of presenilin. The *Drosophila* genome encodes a single *presenilin* gene (*Psn*) [23] circumventing genetic redundancy. All the components of the γ-secretase complex are conserved in flies [24] as is the proteolytic specificity and function of this complex [25], [26]. Since flies do not generate Aβ peptides *Psn* function can also be studied without the confounding impact of Aβ deposition. In addition, studies performed in our laboratory, as well as others, have implicated *Drosophila Psn* function in synaptic plasticity [27], [28], as well as learning and memory [27], [28] further demonstrating that *Drosophila* and mammalian presenilin function is highly conserved and that *Drosophila Psn* is required for processes that are affected in AD.

Here we demonstrate that *Psn* expression in primary *Drosophila* cholinergic neurons causes deficits in intracellular calcium stores. Importantly, these deficits occur independent of Aβ generation. We also describe a novel genetic, physiological and physical interaction between *Psn* and *calmodulin* (*cam*), a key regulator of intracellular calcium homeostasis. Specifically, we show that *Psn*-induced deregulation of internal calcium stores can be suppressed by the loss of a single copy of *cam*. Finally, we also present evidence that Psn and Cam physically interact. Taken together our data support a model whereby presenilin plays a role in regulating intracellular calcium stores that may be influenced by its interaction with Cam and demonstrate that *Drosophila* can be used to study the link between presenilin and calcium deregulation.

## Materials and Methods

### Fly stocks

Flies bearing both a UAS-wild type *Drosophila presenilin* (*UAS-Psn^WT^*) transgene as well as the *cut-GAL4* driver were recombined onto the same third chromosome (*cut-GAL4,UAS-Psn*, referred to from here on in as *cut-Psn*). *cut-Psn* flies were then crossed at 29°C to flies bearing either a P-element insertion in the *Cam* gene (*Cam3909* characterized elsewhere as a recessive hypomorph) [29], [30] or a *Cam* null line (*Camn339*) [31]. The genetic interaction of *Cam* and *Psn* at the wing margin was confirmed by the chi-squared (χ2) 2×2 table method using Statistica software. For the calcium analysis, full-length wild type *UAS-PsnWT* [32] or FAD-M146V mutant (*UAS-PsnFAD*) [33] *Drosophila Psn* transgenes, both on the third chromosome, were crossed at room temperature to flies bearing both a *Cha-GAL4 and UAS-GFP* transgene [34]. Lines bearing both the *Camn339* allele as well as the *UAS-PsnM146V* (*UAS-PsnFAD*) were generated and crossed to the *Cha-GAL4* line described above to assess the physiological interaction between *Cam* and *Psn*.

### Fly imaging

Whole mount images of the pupal CNS were captured using Zeiss LSM 5 Pascal laser-scanning confocal microscope using a 20X objective. Whole mount images of fly wings were generated using the Zeiss Mirax Scan digital imaging platform.

### Cell culture, calcium imaging and analysis

Primary culture, calcium imaging and subsequent analysis were performed according to previously published methods [35], [36] with the following amendments. After baseline recordings were established in four day old primary cultures, the cultures were washed three times over a 200 second interval in HBSS media [35] containing zero calcium, 2 mM EGTA and 4 mM MgCl2. HBSS media containing 5 µM ionomycin (Sigma) was then added to the cultures and the area under the curve was calculated using Mini-Analysis (Synaptosoft) to estimate intracellular calcium content. At least six independent cultures generated on at least three different culturing days were analyzed for each experimental variation. From each neuronal culture, approximately fifteen cells were selected based on positive GFP expression (indicating expression of the cholinergic *Cha-GAL4* driver). Each genotype was coded during analysis and not decoded until all analysis was completed. Statistica software was used for all statistical analysis. Kolmogrov-Smirnov test was used to analyze raw data distribution. Since the raw data of both the resting calcium and ionomycin response measurements was not normally distributed the non-parametric Kruskal-Wallace ANOVA of ranks followed by Mann-Whitney pair-wise comparisons was used to analyze both the calcium baseline and ionomycin responses.

### Binding Assay and Western Analysis

*Drosophila* S2 cells were maintained at room temperature in Schneider's media supplemented with 10% FBS. A construct containing full-length, wild type *Drosophila Psn* under the control of the actin promoter was used to transfect a total of approximately 1×10^7^ S2 cells using the Cellfectin reagent (Invitrogen). 48 hours post transfection, microsomal cell fractions were generated as follows: cells were washed in cold PBS followed by re-suspension in 1.35 mL of 20 mM Tris pH 7.4 with protease inhibitors and then sheared through a 25 gauge needle with subsequent sonication (3×30 seconds on ice). Lysates were then incubated on ice for 15 minutes and spun at 1230× G for 25 min at 4°C. The supernatant was collected and spun further at 100,000× G for 45 min at 4°C. The microsomal pellet was re-suspended in 1 mL of 50 mM Tris pH 7.5 plus protease inhibitors and pre-cleared with 100 µL of agarose beads (Sigma) (pre-washed in 50 mM Tris pH 7.5 buffer) by rotating for 1 hr at 4°C. The sample was then split in half and incubated with either 25 µL of Cam-beads (Sigma) or 25 µL of beads alone (Sigma) for four hours at 4°C. Beads were then washed three times with 50 mM Tris pH 7.5 buffer, allowing beads to settle by the force of gravity in between each 10-minute wash. Finally, bound proteins were eluted using 50 µL of 2× loading buffer with DTT. Western analysis was performed using a rabbit polyclonal antibody raised against the N-terminal portion of the wild type *Drosophila* Psn protein [32] used at 1∶1000 and incubated over-night at 4°C. The anti-calmodulin antibody (Zymed) was used at 1∶1000 over-night at 4°C.

## Results

### *Psn*-induces deficits in intracellular calcium stores

To investigate the effect of *Psn* expression on intracellular calcium dynamics in a cell type relevant to AD, we chose to focus on cholinergic CNS neurons, as their loss is a prominent feature in AD brains [37]. In the *Drosophila* CNS the main excitatory information is provided by excitatory cholinergic information. In addition, *Drosophila* is a genetic model where it is possible to direct expression of different genes in specific cell types. As pupal *Drosophila* CNS neurons are particularly amenable to culturing and evaluation of calcium dynamics, we decided to investigate intracellular calcium dynamics in primary pupal *Drosophila* CNS culture.

**Figure 1:**
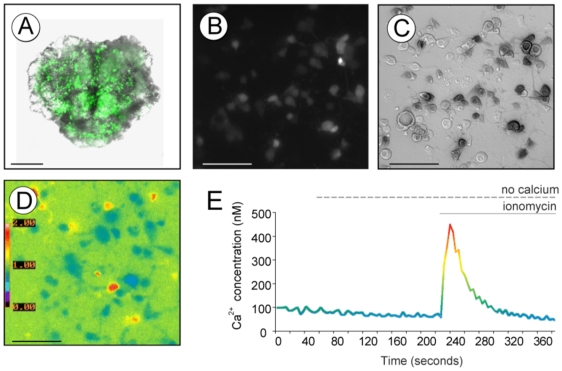
Evaluation of calcium content in internal stores in *Drosophila* primary neuronal cultures. A) Whole mount *Drosophila* CNS 56–72 hour post pupariation expressing GFP in cholinergic neurons driven by the *Cha-GAL4* driver (100 µm scale bar). B) Field of dissociated primary culture showing cholinergic pupal neurons expressing GFP (50 mm scale bar). C) DIC image of primary pupal cultures overlayed with an inverted image showing GFP fluorescent signal in cholinergic neurons (50 mm scale bar). D) Pseudo-colored representation of intracellular calcium concentration in Fura-2 loaded neurons. Cells showing a red hue indicate high intracellular calcium levels while cell bodies in blue represent low calcium levels (50 mm scale bar). E) Over time Fura-2AM measurements can be translated into estimates of real calcium levels. Trace here illustrates results obtained in a typical experiment, where after recording in basal resting conditions, cells are exposed to ionomycin in absence of external calcium.

The *Cha-GAL4* line used in our studies contains a *UAS-GFP* transgene [34] enabling us to specifically select cells expressing *Psn* for calcium analysis (Fig. 1A–C). Calcium dynamics were measured using the calcium binding Fura-2AM fluorescent dye (Fig. 1D). Plotted over time, Fura-2 measurements reveal a calcium trace that can be used to determine resting cytosolic calcium levels as well as calcium movement from internal stores into the cytoplasm (Fig. 1D). Since *Psn* has been shown to impact the calcium content of more than one internal store [10] we chose to measure the release of calcium from all internal stores using the calcium ionophore ionomycin in a recording solution that does not contain calcium (zero extracellular calcium) (Fig. 1E). Previous studies have shown that ionomycin treatment depletes intracellular calcium stores in *Drosophila* cells [38]. We found that ionomycin treatment causes a rapid increase in cytosolic calcium concentration during the initial release of calcium from internal stores as can be seen in Figure 1E. Intracellular calcium levels gradually return to baseline as internal stores are emptied and the calcium is extruded from the cell.

**Figure 2:**
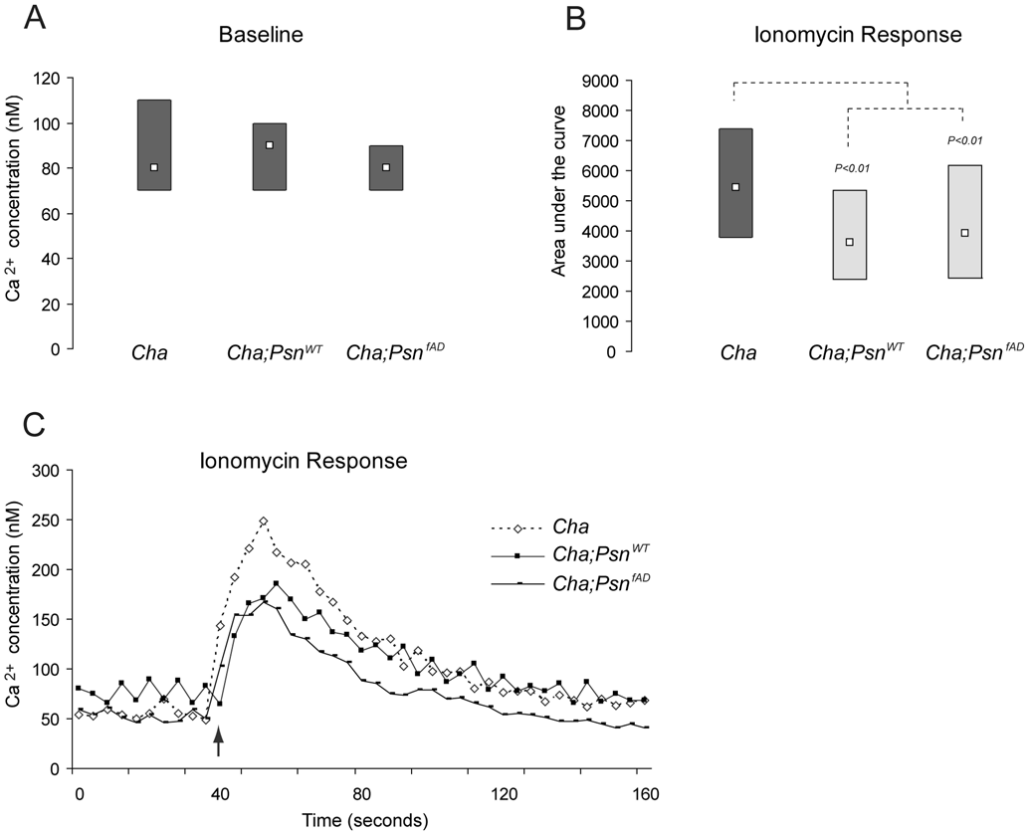
Calcium content in internal calcium stores is affected in cholinergic neurons expressing *Psn*. A) Expression of wild type or FAD-mutant preseniline protein in cholinergic neurons (*Cha;Psn^WT^* and *Cha;Psn^FAD^*, respectively), does not affect basal calcium levels compared to control strain (*Cha*). B) Expression of *Cha;Psn^WT^* or *Cha;Psn^FAD^* results in decrements in intracellular calcium stores. Data are represented as modified box-whisker plots with the median indicated by the smaller white box and the 25 and 75 percent quartiles indicated by the lower and upper margins of the large grey boxes, respectively. Each box represents recordings from cultures generated from at least six independent brains, cultured on at least three independent culturing days. The area under the response curve was calculated from baseline to the point of 50 percent return to baseline in neurons treated with 5 µM of ionomycin. C) Calcium release induced by the application of 5 µM ionomycin (arrow). Each trace represents a response from a single representative cell for each genotype.

The cholinergic *Cha-GAL4* driver was used to drive expression of either wild type (*Psn^WT^*) or FAD-mutant (*Psn^FAD^*) *Psn*. The specific *FAD-Psn* mutant used is a methionine to valine substitution at amino acid 146. Analysis of basal calcium recordings revealed no significant differences between neurons expressing wild type (*Cha;Psn^WT^*, median = 90 nM Ca^2+^) or mutant (*Cha;Psn^FAD^*, median = 80 nM Ca^2+^) *Psn* relative to *Cha-GAL4* controls (*Cha*, median = 80 nM Ca^2+^) suggesting that *Psn* expression is not overtly toxic to these cells (Fig. 2A). Next, we determined whether *Psn* expression could impact calcium levels within internal calcium stores. When cells in culture were exposed to ionomycin under zero extracellular calcium conditions expression of both wild type (*Cha;Psn^WT^* P<0.01, median = 3597.780 nM•s) as well as mutant (*Cha;Psn^FAD^* P<0.01, median = 3926.490 nM•s) *Psn* caused a significant decrease in internal calcium stores relative to controls (*Cha*, median = 5438.02 nM•s). There was no significant difference in internal calcium stores between neurons expressing wild type or FAD-mutant *Psn* (Fig. 2B & C).

### Loss-of-function mutations in *Cam* suppress *Psn-*induced wing scalloping

Recently, we reported that several known regulators of calcium homeostasis suppressed *Psn*-induced phenotypes [39]. Briefly, loss-of-function alleles generated by P-element insertions in the genes encoding the Ryanodine receptor (*Rya-r44F*), calcium binding protein (*CBP*) as well as calmodulin (*Cam*) suppressed the penetrance of either a wing scalloping or thoracic bristle phenotype induced by *Psn* expression. *Psn* has previously been shown to physically interact with, and impact the activity of, the RyR in vertebrates thus demonstrating that our screen could identify true Psn interactors. *Cam* is a calcium signal transducer that activates various enzymes (40) and modulates the activity of various ion channels, including the RyR [41] and IP3R [17]. To date, an interaction between *Psn* and *Cam* has not been described, however, it could represent an important mechanism for regulating intracellular calcium stores.

**Figure 3:**
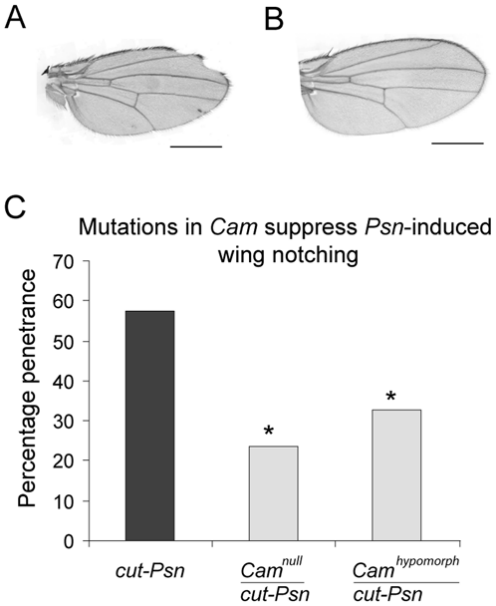
*Psn*-induced wing notching is suppressed by loss-of-function mutations in *Cam*. A) Overexpression of wild type *Psn* under the control of *cut-GAL4* induces a wing notching phenotype in flies (500 µm scale bar). B) The loss of a single *Cam* allele suppresses the *Psn*-induced wing phenotype (500 µm scale bar). C) Quantification of the penetrance of *Psn*-induced wing notching phenotype and the suppression of this phenotype by two loss-of-function mutations in *Cam* (*Cam* null and *Cam* hypomorph). Penetrance was scored based on the presence of at least one wing margin notch. Asterisks denote significant differences in expected penetrance relative to the original *cut-Psn* recombinant as determined by the χ^2^ test.

To confirm that *Psn* and *Cam* genetically interact we generated a recombinant transgenic line, which carried both a wing margin-GAL4 driver (*cut-GAL4*) as well as a UAS-wild type *Psn* transgene (*cut-Psn*). Overexpression of *Psn* at the wing margin gave rise to a wing scalloping phenotype with 58% penetrance (*cut-Psn* Fig. 3A & C). Of note, others have shown that loss of *Psn* function also results in wing scalloping [26]. Overexpression of *Psn* in *Drosophila* is believed to give rise to dominant negative effects since overexpression phenocopies *Psn* loss-of-function [26], [33]. Flies bearing either a P-element insertion in *Cam* (characterized elsewhere as a hypomorphic *Cam* allele) [29] or an imprecise excision in *Cam (Cam^null^* [42], which was not used in the original screen) were crossed to *cut-Psn* recombinant flies. Both the *Cam* hypomorph (33% penetrance, χ^2^ = 9.9, P<0.05) as well as the *Cam* null (24% penetrance, χ^2^ = 10.49, P<0.05) significantly suppressed the penetrance of the *Psn*-induced wing scalloping phenotype (*cut-Psn* penetrance  = 58%, Fig. 3B & C) thereby confirming that *Psn* and *Cam* genetically interact.

### *Cam* suppresses *Psn*-induced deficits in intracellular calcium stores content

**Figure 4:**
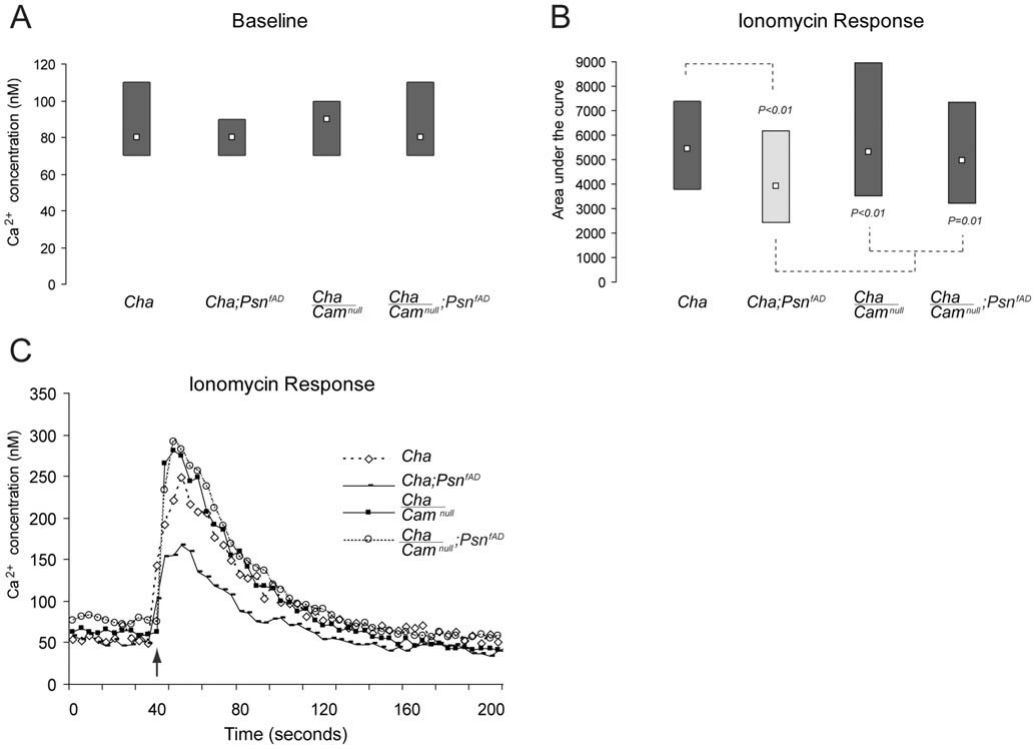
*Psn*-induced effects on intracellular calcium stores are suppressed by a loss-of-function mutation in *Cam*. A) Resting calcium levels are not affected by the loss of a single *Cam* allele either in a wild type (*Cha/Cam^null^*) or in a *Psn* mutant background (*Cha/Cam^null^; Psn^FAD^*), compared to their respective controls (*Cha* and *Cha;Psn^FAD^*, respectively). B) Loss of a single allele in *Cam* suppresses *Psn*-induced deficits in calcium stores content (*Cha/Cam^null^;Psn^FAD^* compared to *Cha;Psn^FAD^*, P = 0.01). Neurons were treated with ionomycin and the area under the curve was calculated from baseline to the point of 50 percent return to baseline. Data are represented by modified box-whisker plots as described in Figure 2. Light grey boxes identify groups that are statistically different from the *Cha-GAL4* control. Each box represents recordings from cultures generated from at least six independent brains, cultured on at least three independent culturing days. C) Calcium release induced by the application of 5 µM ionomycin (arrow). Each trace represents a response from a single representative cell for each genotype.

Given that *Cam* is known to play an important role in the regulation of intracellular calcium levels, we wanted to examine whether *Psn* and *Cam* physiologically interact to regulate internal calcium stores. For this purpose we decided to focus on mutant FAD-*Psn* rather than wild type *Psn* since expression of both transgenes gave rise to similar deficits in internal calcium stores and because the interaction of *Cam* with an FAD-mutant phenotype would be more relevant to AD etiology. The resting calcium levels in *Cha/Cam^null^* transheterozygotes (*Cha/Cam^null^* median  = 90 nM Ca^2+^) were not significantly different to resting levels in *Cha-GAL4* alone (*Cha* median  = 80 nM Ca^2+^) (Fig. 4A). Likewise, resting calcium levels in neuronal cultures generated from flies expressing *FAD-Psn* in cholinergic neurons with only a single functional *Cam* allele (*Cha/Cam^null^;Psn^FAD^* median  = 80 nM Ca^2+^), also appeared normal relative to *Cha-GAL4* controls (Fig. 4A). We then exposed these cells to ionomycin in the absence of external calcium to evaluate internal calcium stores. Importantly, loss of a single *Cam* allele alone (*Cha/Cam^null^*) did not alter the calcium content of intracellular stores relative to *Cha-GAL4* control cells (Fig. 4B). However, as can be seen in Figure 4B, loss of a single *Cam* allele did suppress the *Psn*-induced calcium store decrements (*Cha/Cam^null^;Psn^FAD^*, median  = 5322 nM•s, P = 0.01) otherwise observed in neurons expressing FAD-*Psn* with two functional copies of *Cam* (*Cha;Psn^FAD^*, median  = 3926 nM•s) (Fig. 4B & C). There was no significant difference in ionomycin-induced calcium release between *Cha/Cam^null^;Psn^FAD^* and *Cha-GAL4* neurons. Thus, loss of a single *Cam* allele can also suppress *Psn*-induced calcium stores deficits.

### Psn and Cam physically interact

**Figure 5:**
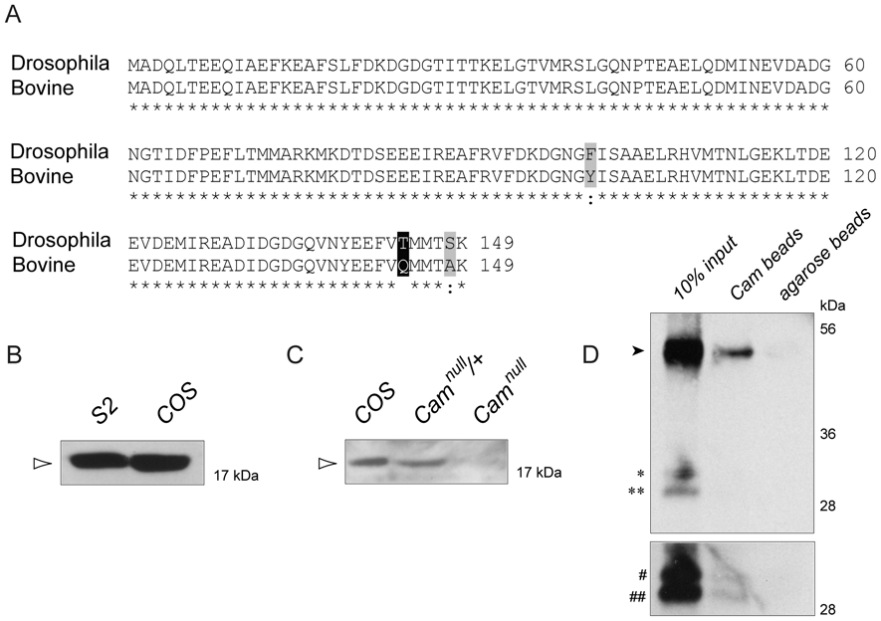
Cam binds to full-length as well as the N-terminal fragment of Psn. A) Sequence alignment of Bovine as well as *Drosophila* Cam demonstrates the high degree of conservation of Cam between the two species. Identical sequence homology (asterisk), similar amino acids (light grey box) and the one different amino acid (black box) are highlighted. B) Antibodies raised against bovine Cam recognize mammalian Cam (arrowhead) in COS cells as well as *Drosophila* Cam in S2 cells, highlighting the high degree of conservation of this protein. C) The specificity of the Cam antibody was confirmed using lysates generated from *Cam^n339^* homozygous null larvae (*Cam^null^*) where the band corresponding to Cam is absent, as opposed to lysates generated from *Cam* heterozygotes (*Cam^null^/+*) and mammalian control COS cells (COS). D) Equal amounts of protein were incubated with either Cam-beads or beads alone. Full-length (black arrow head) and the N-terminal fragment of Psn in a short (asterisk) or long (pound) exposure were pulled down by Cam-beads but not beads alone. Two N-terminal Psn bands indicated in the gels correspond to two isoforms generated by alternative splicing of Psn.

We then sought to determine if the ability of *Cam* to suppress *Psn*-induced deficits in intracellular calcium stores was due to a direct versus indirect interaction between the two proteins. Presenilins are known to physically interact with other calcium sensing proteins. For example, mammalian presenilin 2 has been shown to bind to the EF-hand motif of sorcin [43]. Since Cam contains four EF-hand motifs, we reasoned that Psn may physically interact with Cam as well. Cam is highly conserved among species (Fig. 5A–C). This conservation has enabled us to take advantage of the commercial availability of agarose beads covalently bound to bovine Cam to perform binding assays. Lysates were generated from *Drosophila* S2 cells transfected with full-length wild type *Psn*. Equal amounts of protein were incubated with either beads alone or beads covalently bound to Cam. Normally, full-length Psn is rapidly processed into N- and C-terminal fragments hence full-length Psn is rarely observed. However, since Psn processing is dependent on limiting factors, when Psn is overexpressed the full-length holoprotein accumulates (Fig. 5D black arrow head) while the N- and C-terminal fragment levels remain unaltered. The two N-terminal Psn bands (Fig. 5D asterisk) correspond to two different isoforms resulting from alternative splicing [23]. Western analysis revealed that indeed, full-length Psn, and to a lesser extent, the cleaved N-terminal fragment bound to Cam-beads but not to beads alone (Fig. 5D).

## Discussion

Although the specific cellular mechanisms remain uncertain, increasing evidence suggests that presenilins play an important role in regulating intracellular calcium dynamics. We have investigated *Psn* function in the context of intracellular calcium homeostasis using the *Drosophila* CNS as a model system. Our data demonstrates that expression of wild type or FAD-mutant *Psn* in *Drosophila* cholinergic neurons results in cell-autonomous deficits in calcium stores. Decrements in calcium stores attributed to wild type or mutant *Psn* expression have been documented in human cells as well as mouse models [10], [13], [17], [44]. To date, most studies in *Drosophila* have focused on the role of *Psn* in Notch signaling during development. Our data clearly demonstrate that *Drosophila* can be used as a model to study additional functions of *Psn* and specifically, its role in the regulation of intracellular calcium dynamics. Importantly, unlike mammals *Drosophila* do not generate Aβ. Hence, in our model system, any effects on internal calcium stores can be attributed entirely to presenilin and is not confounded by the production of Aβ peptides.

Interestingly, we found that expression of either wild type or FAD-mutant *Psn* gave rise to deficits in intracellular calcium stores. Ot OOhers have also reported intracellular calcium deregulation in cells expressing either wild type or FAD-mutant *Psn* [13], [17]. We believe that the calcium decrements we observe are due to a loss of *Psn* function since previous studies have shown that overexpression of wild type or FAD-mutant *Psn* in *Drosophila* gives rise to dominant negative effects [33]. The mechanistic nature of the dominant negative effect is not known but it may involve negative repercussions of holoprotein accumulation within the ER or competition for limiting factors that enable the formation of functional γ-secretase complexes. Importantly, there is mounting evidence suggesting that loss of *Psn* function is responsible for some aspects of FAD pathogenesis. For example, conditional knock out of both *presenilin 1/2* phenocopies AD-like symptoms including, learning and memory impairments as well as progressive neurodegeneration [45]. Notably, these *Psn*-induced phenotypes were observed in the absence of Aβ deposition. Another group has also demonstrated memory loss and degeneration associated with loss of both *presenilins* in the mouse brain, once again, in the absence of Aβ generation [46]. In accordance to these findings, we have previously shown that loss of *Psn* function in *Drosophila* results in defects in synaptic plasticity and learning [27]. In addition, in both *Drosophila* [47] and *C. elegans* [48], [49], FAD-mutations in *Psn* fail to completely rescue loss of wild type *Psn* function. Since decrements in internal calcium stores have been documented in mammalian PS1/PS2 null cells [50] our data are consistent with a dominant negative effect arising from *Psn* overexpression.

A great deal of effort has been made in several model systems to resolve how presenilin function impacts intracellular calcium dynamics. The results of one study suggested that wild type, but not FAD-mutant presenilin, exhibits passive calcium channel activity [22]. Since our results indicate that both wild type and FAD-mutant Psn cause decrements in intracellular calcium stores our data do not support a putative passive calcium channel function for wild type Psn in *Drosophila* [22]. Presenilins are not known to bind calcium directly hence their influence on intracellular calcium signaling may be mediated by interactions with calcium binding proteins and indeed, presenilins have been shown to bind several calcium binding proteins [43], [51], [52]. Using two independent loss-of-function alleles in *Cam* we have confirmed that loss-of-function mutations in *Cam* suppress a *Psn*-induced wing phenotype. The mechanism for this suppression may also involve intracellular calcium stores. Wing scalloping is a classic *Notch* loss-of-function phenotype. Although we do not have direct evidence to suggest that *Psn*-induced wing notching is attributed to disruptions of intracellular calcium at the wing margin, it is known that Notch proteolysis and activity is impacted by changes in internal calcium stores. For example, loss-of-function mutations in the *Drosophila* calcium-ATPase gene *Ca-P60A* have been shown to cause aberrant Notch trafficking and secretion due to alterations in internal calcium stores [53]. Hence, it is conceivable that *Psn*-induced deregulation of internal stores is responsible for the observed Notch phenotypes.

Since *Psn* has been linked to calcium deregulation and *Cam* is an important player in intracellular calcium homeostasis we further investigated the genetic interaction between *Psn* and *Cam* in a cellular context relevant to AD. Using primary *Drosophila* cholinergic neurons we found that loss of a single functional *Cam* allele suppressed calcium store deficits otherwise observed with the overexpression of FAD-mutant *Psn*. Furthermore, we showed that Cam physically interacts with both full-length as well as the N-terminal fragment of Psn, albeit to a lesser extent relative to the holoprotein. A physical interaction between Cam and Psn has previously been postulated using a Calmodulin Target Database, which identified putative Cam binding sites in presenilin 1 and 2 [18]. In fact, this database identified putative Cam binding sites in all of the components of the γ-secretase complex [18]. Since Cam binds both full-length as well as the N-terminal fragments of Psn, Cam may play a role in regulating Psn proteolysis or protein stability. Cam activity has previously been implicated in regulating the stability and proteolysis of other integral membrane proteins [54]. Interestingly, others have shown that calmodulin RNA levels can be impacted by FAD-*Psn* expression [55]. We have been unable to detect any effect of loss of a single Cam allele on the FAD-presenilin protein levels (Figure S1). Since both presenilin and calmodulin are known to regulate ER calcium channel, it remains possible that these two proteins interact at the level of intracellular calcium channel regulation.

Given that Cam activity is known to play a role during learning and memory, apoptosis, as well as tau phosphorylation, the interaction between *Psn* and *Cam* may be very relevant to AD pathogenesis. Only by characterizing how normal and aberrant *Psn* activity impact calcium homeostasis can we begin to resolve how this cellular process contributes to AD pathogenesis.

## Supporting Information

Figure S1 FAD-mutant Psn protein levels. A) Western analysis of lysates generated from adult fly heads. Loss of a single Cam allele does not appear to alter the level of Psn holoprotein (single asterisk) in flies expressing FAD-mutant Psn. N-terminal Psn fragment levels (double asterisk) also appear unaltered by the loss of a single Cam allele. Actin protein levels serve as loading control (solid black arrow head in both lanes). B) Densitometry analysis of band intensity for full-length presenilin (B) or the N-terminal fragment (C) normalized to actin levels determined that loss of a single Cam allele did not alter presenilin protein levels in flies expressing FAD-mutant presenilin. B) & C) represent quantitative results based on the analysis of three independent Westerns.
(0.13 MB TIF)
