# Correction: Intracellular Calcium Deficits in *Drosophila* Cholinergic Neurons Expressing Wild Type or FAD-Mutant *Presenilin*

**DOI:** 10.1371/annotation/f8cecc73-06ec-4bba-b07a-a3bc2a08f4c7

**Published:** 2009-09-24

**Authors:** Kinga Michno, David Knight, Jorge M. Campusano, Diana van de Hoef, Gabrielle L. Boulianne

The third author's name was spelled incorrectly. The correct name is: Jorge M. Campusano. The correct citation is: Michno K, Knight D, Campusano JM, van de Hoef D, Boulianne GL (2009) Intracellular Calcium Deficits in Drosophila Cholinergic Neurons Expressing Wild Type or FAD-Mutant Presenilin. PLoS ONE 4(9): e6904. doi:10.1371/journal.pone.0006904

