# Supplementary figures and images for: Intracellular Calcium Deficits in Drosophila Cholinergic Neurons Expressing Wild Type or FAD-Mutant Presenilin
Source: PLoS One. 2009 Sep 4;4(9):e6904. doi: 10.1371/journal.pone.0006904 (PMC2733141; doi:10.1371/journal.pone.0006904)

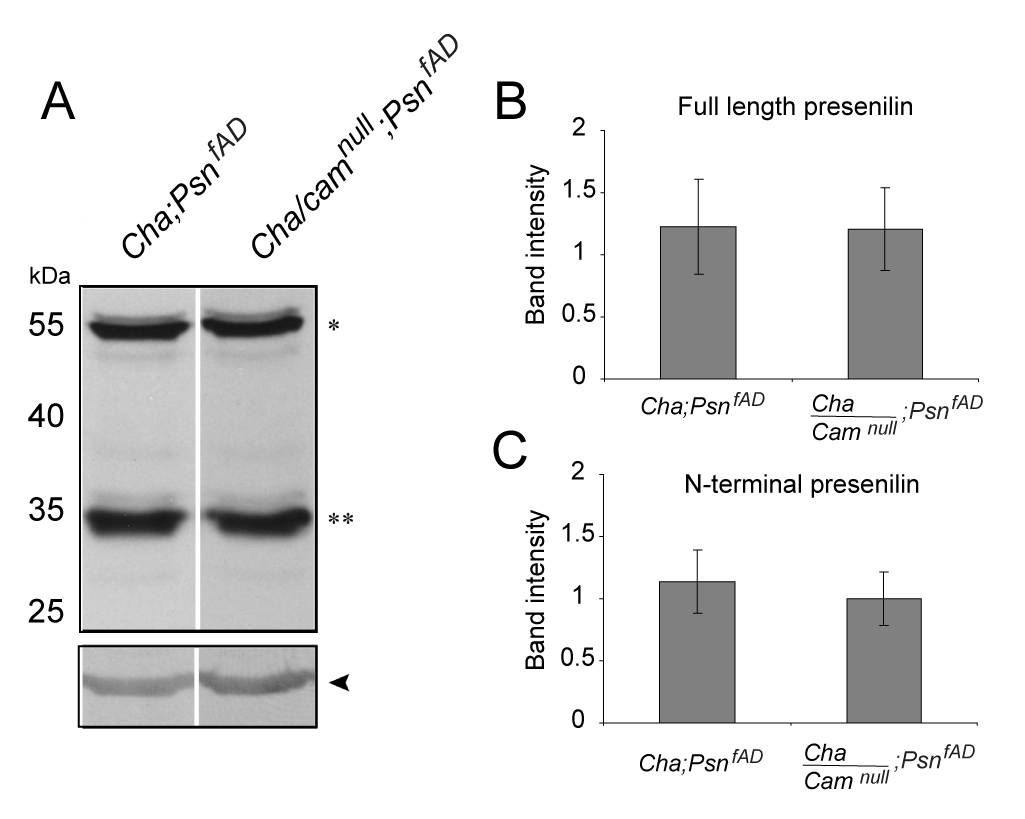

Supplement: Figure S1 — FAD-mutant Psn protein levels. A) Western analysis of lysates generated from adult fly heads. Loss of a single Cam allele does not appear to alter the level of Psn holoprotein (single asterisk) in flies expressing FAD-mutant Psn. N-terminal Psn fragment levels (double asterisk) also appear unaltered by the loss of a single Cam allele. Actin protein levels serve as loading control (solid black arrow head in both lanes). B) Densitometry analysis of band intensity for full-length presenilin (B) or the N-terminal fragment (C) normalized to actin levels determined that loss of a single Cam allele did not alter presenilin protein levels in flies expressing FAD-mutant presenilin. B) & C) represent quantitative results based on the analysis of three independent Westerns. (0.13 MB TIF) [file pone.0006904.s001.tif]
